# Environmental Sustainability Assessment of the European Union’s Capital Cities

**DOI:** 10.3390/ijerph19074327

**Published:** 2022-04-04

**Authors:** Mariusz Czupich, Justyna Łapińska, Vojtěch Bartoš

**Affiliations:** 1 Faculty of Economic Sciences and Management, Nicolaus Copernicus University, Gagarina 11, 87-100 Toruń, Poland; justlap@umk.pl; 2 Faculty of Business and Management, Brno University of Technology, Kolejní 2906/4, Královo Pole, 612 00 Brno, Czech Republic; bartos@vut.cz

**Keywords:** sustainable development, urban environment, city ranking

## Abstract

The growing flow of people into cities causes several challenges for their functioning. This brings the need to ensure, for example, efficient transport, sustainable waste, and appropriate energy policy. Particularly capital cities are exposed to the above-mentioned risks, due to their large numbers and densities of inhabitants. Therefore, the state of the environment in cities should be monitored systematically. The research aim of this article is to evaluate the level of environmental sustainability in capitals of European Union countries. A synthetic indicator was made up of diagnostic variables, using quantitative and qualitative indicators relating to the quality of the environment. Based on the ranking, results reveal that Europe is spatially divided according to the level of environmental quality. The best results were achieved by capital cities of the northern European countries. The analysis presented here has some application potential. It can serve to identify challenges to improving the quality of the environment, and to raise public awareness of the importance of changing individual behaviour (e.g., use of public transport).

## 1. Introduction

Currently, the importance of cities as the main centres of socioeconomic life is increasing. On the one hand, they are becoming the main places where people settle. Indeed, today 55% of the world’s population lives in cities, and by 2050 this share will reach 70% [1,2]. In the European Union, these rates are even higher, at 70% and 80%, respectively [3]. On the other hand, cities generate more than 80% of global GDP. Following these processes, cities and the entities within them consume two-thirds of global energy and emit more than 70% of greenhouse gases [4].

The increase in population density and pollution has several consequences for the functioning of cities. These concern the decrease in efficiency of public services related to public transport, waste, energy, administration, technical and social infrastructure, etc. [5,6]. Achieving greater inclusion, sustainability, and resilience is also becoming an issue. In the environmental field, the growth of the urban population implies problems related to climate change, energy consumption, greenhouse gas emissions, and environmental degradation.

Faced with the above challenges, the international community has undertaken a number of initiatives aimed at balanced social development, economic growth, and environmental protection, including the 2030 Agenda for Sustainable Development [7], the New Urban Agenda [8], and the Paris Agreement [9]. They are the consequence of Europe’s longstanding active role in many agreements and undertakings related to sustainable development, such as the 1987 Montreal Protocol, the 1992 Rio World Summit, the 1997 Kyoto Protocol, and the 2002 Johannesburg World Summit [10].

European cities have made a commitment to reduce greenhouse gas emissions and reduce the consumption of fossil fuels. They are assisted in this by funds under the European Cohesion Policy, which support the production of renewable energy and improvements in energy efficiency. Cities are considered the spatial units that can most influence the transition to a ‘low-carbon’ economy, since their efficiency in energy use and land use is higher than other areas [11]. This is due, among other things, to the fact that most people live in apartments or terraced housing, which are more energy efficient than free-standing houses. Moreover, short distances favour walking and cycling and the use of urban transport, which generate less CO₂ than cars.

The condition of the natural environment in cities is an interesting and very complex issue. It depends on different factors, including transport, air quality, water quality, green areas, noise levels, and cleanliness. Each of these areas is identified by appropriate indicators. The factors most commonly used in environmental studies of cities are air quality (PM₁₀ concentration), energy (renewable energy resources), and climate (CO₂, ecological footprint, greenhouse gas emissions) [12]. The impact of individual factors on the final results varies considerably. In some analyses, air quality and sanitation show the most significant impact [13]. In others, however, the focus is on green spaces and water resources [14].

Despite the fact that European cities score better than cities from other continents in environmental rankings, they still face many barriers to development. Indeed, the biggest challenges are municipal waste generation and greenhouse gas emissions [15]. This implies a transformation of political, economic, and social actions. The actions of municipal authorities and economic organisations are supported by national and EU public funds. However, citizens, often on their own, must challenge the change of heating systems, modes of transport, and investments in renewable energy. In addition, their daily behaviours regarding waste separation, electricity and water consumption, and the purchase of products with a lower environmental footprint, are crucial to the health of the environment. The environmental awareness of Europe’s inhabitants varies widely spatially. It is the result of many factors. Interestingly, one of them is the quality of government. Indeed, it turns out that countries with a higher level of quality of government have a higher propensity of citizens who behave pro-environmentally [16].

The transformation of a city to a sustainable model is a long-term process, and the results depend on the involvement of many stakeholders [17,18]. On the one hand, it is necessary to change the way people consume and their daily behaviours to minimise the negative impact on the environment [19]. On the other hand, however, political support is required. City authorities should set appropriate laws and rules, but also cooperate with the private sector to improve the capacity to collect and recycle waste, facilitate distributed generation of renewable energy, build energy efficiency, and ensure the cleanliness of all material flows into and out of the city [20,21]. Broad alliances and networks between different social, economic, and political actors are needed to gain support, acceptance, and legitimacy for green vision activities [22,23]. Clean energy transitions in cities are also related to building infrastructure based on clean energy, renewable energy use, air quality data management, energy consumption, and traffic patterns [24,25,26]. The most demanding element in terms of financial investment is infrastructure, including microgrids and smart grids, new waste management and recycling facilities, and mass transit systems. 

The level of environmental sustainability is the result of the activity of all urban actors. It demonstrates mutual understanding and cooperation for a clean environment or the lack thereof. The climate in European capitals is undergoing rapid change. Studies confirm an increase in magnitude of heat waves and a decrease in magnitude of cold spells in these units [27]. This demonstrates the need for continuous environmental monitoring to identify risks and implement countermeasures.

The research aim of this article is to evaluate the level of environmental sustainability for capitals in European Union countries. In this study, a set of diagnostic variables was selected and used to develop a ranking of European Union capital cities according to their level of environmental sustainability. The study made it possible to group the analysed cities into four groups according to the advancement of this level. The synthetic measure method was used. The idea behind this method is universal and it can be used to estimate the degree of environmental sustainability of other spatial units as well.

The following research questions were formulated in the study:

Q1: Which environmental indicators are used in comparative analyses of spatial units?

Q2: Which European capital cities present the highest level of environmental sustainability and which ones present the lowest?

Q3: Does the differentiation of European Union capital cities by level of environmental sustainability show specific spatial relationships?

Presenting the sustainability situation of European capitals in the form of a ranking has several advantages: (i) it allows us to identify which cities face the greatest challenges in improving environmental quality and the need for greater involvement in this process by city authorities, scientists, businesses, social organisations, and residents themselves; (ii) it informs about the efficiency of city authorities in sustainable management and the importance (role) of environmental aspects in the development strategies implemented in capitals so far; (iii) it raises public awareness of the importance of changing individual behaviour (e.g., reducing driving) for the good of the environment and society at large. 

In the first section of this article, a brief overview of sustainable development measurement methods is presented, focusing on indices for assessing countries and cities. The analysis covers both sustainable development rankings in the holistic approach, and those relating only to the environmental dimension. This section also provides substantive justification for the use of indicators related to, among others, air pollution and green spaces in environmental analyses. The second section describes the methodology used in the empirical part of the article. It also presents a description of the diagnostic variables and the standardisation procedure applied. In the next section, empirical findings are presented and discussed. The article ends with general conclusions on environmental sustainability in European capital cities. 

## 2. Rankings and Indicators of Environmental Sustainability 

Measuring the level of sustainable development is complicated by the multiplicity of ways of understanding it. Moreover, it is a long-term process involving desired changes and proposals of conduct in many aspects while, importantly, not defining the final vision. Complications are caused by multidimensionality of processes and phenomena forming sustainable development, their dynamism, and cause-effect relations. All this makes the definition of measurement methods and appropriate selection of indicators a difficult task. The development of the concept of sustainable development since the 1960 s has resulted in a growing number of methodologies, models, approaches, and appraisals for assessing sustainability. It is estimated that at present there may be several hundred different instruments for assessing sustainability [28,29]. 

Sustainability assessment tools can be divided into three main categories: monetary, biophysical, and indicator-based [30]. The first category is based on models of human behaviour and estimating their impact on certain benefits, costs, and utility in terms of specific phenomena and processes. Monetary tools include cost-benefit analyses used to assess the ecological advantages and disadvantages of decisions. The assessment takes place by identifying the benefits and costs of a decision to determine its impact on welfare. It can also be used to assess the impact of CO₂ reduction costs on environmental benefits [31]. Biophysical tools, in turn, are used to assign values to objects based on their intrinsic properties. For this purpose, they measure physical parameters of these objects and translate them into a specific unit of measurement. An example of a biophysical tool is the ecological footprint. It uses quantities expressed in physical units to assess the intensity of natural resource management (bio-productive land) [32]. The third category of sustainability assessment tools includes indicator-based ones. These are related to the need to make multiple methodological choices regarding the set of indicators used to weight, normalise, and aggregate. This means that the results of the study largely depend on the research procedure. These tools are used to assess the phenomenon under analysis using several weighted indicators. An example of this type of tool is the composite indicator tool, which is based on normalisation and aggregation of indicators [33]. An alternative is multi-criteria assessment, where no aggregation of indicators is performed [34].

According to Macnaghten and Jacobs [35] in the sustainable development model, economic well-being as a component of quality of life is limited by environmental conditions. Hence, the environment is a prerequisite for the occurrence of other types of progress and therefore, the term sustainability is mainly associated with the environment [36]. The measurement of the environmental aspect may emphasize different selective emphases (e.g., resources), or it may deal with the environment in a holistic way. Thus, the available methods for assessing the environmental sustainability of spatial entities, such as countries and cities, can be divided according to measurement categories: indicators/indices (Sustainable Development Indicators, Environmental Pressure Indicators, The Dashboard of Sustainability), Resource Availability Assessment (Ecological Footprint, Water Footprint), Material and Energy Flow Analysis (Substance Flow Analysis, Physical Input-Output Tables), Life-Cycle Assessment (Life-Cycle Sustainability Analysis) [37].

Indicator-based measurement methods have become quite popular in recent years [38]. There is a very rich set of rankings of countries according to synthetic indicators. For instance, the SDG Index ranks United Nations countries according to their performance on the 17 goals set out in the 2030 Agenda for Sustainable Development [39]. A slightly broader approach is presented by the Green Growth Index, which, in addition to the Sustainable Development Goals, analyses the Aichi Biodiversity Targets and the achievements of countries under the Paris Climate Agreement [40]. The Global Green Economy Index, on the other hand, uses 20 quantitative and qualitative indicators divided into four areas to rank countries: leadership and climate change, efficiency sectors, markets and investment, and the environment [41]. At the country level, rankings related only to the environmental dimension of sustainable development are also published. One of them is the Environmental Performance Index, ranking approximately 180 countries according to environmental health and ecosystem vitality [42,43]. It follows on from an earlier index, the Environmental Sustainability Index (ESI), which was published between 1999 and 2005 through the involvement of Yale University and Columbia University in collaboration with the World Economic Forum and the Joint Research Centre of the European Commission. 

The construction of synthetic indicators is a complex process involving the selection of different methods, tools, techniques, and variables. This may cause various issues of uncertainty due to the selection of data, erroneous data, data imputation methods, data normalisation, standardisation, weighting methods, weights’ values, and aggregation methods [44]. A criticism of many environmental quality assessments also concerns the use of relative measures that rank spatial units according to the value of a particular indicator [45]. Admittedly, they allow identification of changes in a given location or time, but do not use references to a pattern of development. An alternative method is based on using absolute indicators that are compared to an environmental threshold (scientifically defined), which makes it possible to assess the actual state of the urban environment. Lancer and Nijkamp [46] even argue that an indicator does not illustrate sustainability until it is referenced to a reference value, such as a threshold. 

The role of indicators is to assess the effectiveness of policies and to provide guidance for policymaking [47]. It is particularly important to provide decision makers ‘with an evaluation of global to local integrated nature-society systems in short and long-term perspectives to assist them to determine which actions should or should not be taken in an attempt to make society sustainable’ [48].

The possibilities to measure urban sustainability have increased as a result of data collection by international institutions involved in the fight against climate change, such as the European Environment Agency, the World Bank, the European Commission-Eurostat, the OECD, the WHO, or the UN. Relatively new sources of data include the GIS platform to generate maps of noise and pollutant concentrations [49], the Numbeo platform (a global database also including perceptions of air pollution), maps with electric car charging stations, and portals with air measurement station results. 

Many sustainability measurement concepts and data collection procedures have also emerged in connection with EU-funded projects, such as CITYkeys, KITCASP, and ECO-city. 

The methodology for measuring sustainability of cities requires a combination of quantitative and qualitative processes to achieve the accuracy of the quantitative assessment and the comprehensiveness of the qualitative analysis results [50]. This approach is used, among others, by the Green City Index developed by the Economist Intelligence Unit in collaboration with Siemens. The index is based on about 30 indicators divided into eight or nine sections (depending on the region), including land use, transport, water, air quality, and waste management. Quantitative and qualitative indicators are roughly half in each section [51]. To a slightly lesser extent, qualitative indicators are used in the City Blueprint Framework, where much attention is paid to water issues [52]. However, in the European Green Capital ranking the experts of the technical assessment panel evaluate cities on the basis of 12 environmental indicators and measures implemented over the last five to ten years, and short-term and long-term environmental objectives [53]. 

The rankings mentioned above are only concerned with the environmental dimension. Nevertheless, this dimension is also included within broader studies that rank sustainable cities [54,55,56], smart cities [57,58,59,60], or smart sustainable cities [33,61]. In such cases, the environmental dimension is one of several elements considered alongside, for instance, ICT, living, economy, mobility, people, and government. It is important to keep in mind a high quality of life for communities when selecting indicators for this type of measurement, and not to focus only on technology-driven solutions but also to consider non-technological aspects [62].

The above-mentioned rankings are characterised by certain strengths as well as weaknesses [63]. Weaknesses include using rankings not suitable for global benchmarking (European Green Capital Award), lack of data for some indicators (IESE Cities in Motion Index), assigning indicators related to country-level or regional level to city-level (The 2019 SDG Index and Dashboards Report for European Cities; Arcadis Sustainable Cities Index). Caution is therefore needed in interpreting the results; on the other hand, gaps may be created for other such studies.

A list of environmental indicators of selected city rankings produced by private and public organisations is very broad. It can be seen that categories such as air quality, green spaces, energy, waste, and transport tend to dominate.

Air pollution is currently the most significant environmental risk to health, and is considered the second greatest environmental challenge for European countries after climate change [64]. Harmful gases emitted into the atmosphere cause many diseases and consequently increase health care costs, especially among urban populations. They also have an impact on the decrease in work efficiency and the emergence of civilization diseases. They cause damage to buildings, decreased air transparency, and smog problems. Exposure to polluted air of pregnant women causes impacts on fertility, pregnancy, newborns, and children [65]. Harmful gas emissions are largely derived from the structure of the urban area, industrial zoning, and transport roads, where there is a higher concentration of NO₂ and PM10 [66]. The European Commission and European Environment Agency are undertaking several activities related to increasing public environmental awareness and behavioural changes, but also increasing monitoring of pollutants, in particular ozone (O₃), particulate matter (PM), nitrogen dioxide (NO₂), benzo[a]pyrene (Bap), and sulphur dioxide (SO₂) [67].

Air pollution levels can be used in combination with environmental noise to measure urban environmental quality [49]. The City Noise-Air index measures the most annoying aspects of urban life for citizens and has the potential to support decision-making by urban planners and policy makers. Environmental noise itself has a significant impact on health and well-being, causing cardiovascular disease, cognitive impairment, sleep disturbance, tinnitus, and annoyance [68]. It is a result of population growth and urbanization, in addition to roads and related air and rail traffic. It encompasses all sounds that disrupt sleep, concentration, and communication beyond those in the workplace [69].

The impact of noise, pollution, and congestion on health and well-being is not only one experienced by city dwellers. It turns out that an important factor that can have both positive and negative impacts is the physical urban environment in the form of water, waste, and sanitation infrastructure; street design and connectivity; public transport; building materials and design; green spaces and parks; and the urban heat island effect [70]. Of particular importance are green spaces, including trees, parks, nature conservation areas, sports fields, riverbanks, green walls, and alleyways. They generate a range of environmental benefits. Green spaces filter the air and remove pollutants, dampen noise, reduce temperatures in hot weather, and enable groundwater recharge. Furthermore, research findings confirm the positive impact of green spaces on mental well-being [71,72], the capacity for self-regeneration and greater physical activity [73], stress reduction [74], and even income levels of residents [75]. In contrast, the experiences of US and Chinese cities show that open green spaces and areas can be considered as a tool for environmental justice [76]. In poor neighbourhoods, neglected and industrial urban areas, places for recreation, and leisure are created that promote physical activity and public health. It is worth adding that there may, however, be adverse effects of such activities in the form of gentrification of entire areas [77].

Among the proposals for indicators to measure environmental aspects, there are also those that relate directly to the performance of city authorities [78]. These include various aspects of public transport, access to basic services, road, pedestrian and cycling infrastructure, sustainable land use, and re-cultivated green and water elements. 

Urban buildings are also environmentally relevant. Buildings, together with industry and the transport system, are major energy consumers. It is therefore important to determine their efficiency and energy self-sufficiency. Among the recognised certifications for sustainable buildings are LEED (developed in the USA), Green Globes (USA), BREEAM (UK), ATHENA (Canada), BEAT 2002 (Denmark), or CASBEE (Japan). For example, Leadership in Energy and Environmental Design (LEED) is used in the form of total building square meterage that is covered by LEED certification in the Global Green Economy Index ranking [79].

A relatively new type of urban infrastructure is electric car charging stations. Their number is linked to the increasing demand of residents. Studies have confirmed a positive relationship between the share of electric vehicle sales and public charging stations in metropolitan regions [80]. In 2020, Europe had the highest number of electric cars registered in the world, with almost 1.4 million (625 thousand plug-in electric vehicles and 747 thousand battery electric vehicles); this represents an increase of more than 140% compared to 2019 [81]. This increment was mainly due to the adjustment of car manufacturers to the new European Union standards for reduced CO₂ emissions for new cars [82]. Thus, the aforementioned indicator may be related to the greater environmental awareness of urban residents, and to the desire to reduce exhaust emissions.

## 3. Materials and Methods

Given the increasing role of environmental protection in socioeconomic life, the authors attempted to identify the environmental level among the largest cities in European Union countries. Thus, it was decided to identify the factors shaping this level. First, the authors asked the following question: which environmental indicators are used in comparative analyses of spatial units? To select diagnostic variables, existing rankings of cities using environmental indicators were analysed. Attention was drawn to the popularity of certain indicators (e.g., air pollution), their significance for the quality of life of the inhabitants, and the need to combine the quantitative and qualitative dimensions. The set of variables thus selected was used to answer the next research question: which European capital cities present the highest level of environmental sustainability, and which ones present the lowest? EU capital cities are an interesting subject of research, as they are usually highly populated areas with the highest densities where environmental threats accumulate. Furthermore, they differ significantly in terms of the specific behaviour of individual nationalities, attitudes towards ecology, or the efficiency of municipal authorities in the field of green initiatives. The results made it possible to determine the spatial dependencies related to the level of environmental sustainability, which provided an answer to the third research question.

The environmental sustainability of cities is a complex phenomenon. The concept of a complex phenomenon is defined in literature on the subject as follows [83]: a complex phenomenon is an abstract entity illustrating the qualitative state, directly unmeasurable of the real objects, described by several variables (greater than one), which are called diagnostic variables.

Complex phenomena are described by at least two characteristics, which may have different titres and different orders of magnitude. Multivariate methods are used to analyse such phenomena, and are used by researchers in many fields, including economic sciences, but also sociology, medicine, demography, and others [84,85,86,87,88].

Urban environmental sustainability as a multidimensional construct is a typical example of a complex phenomenon. This means that to assess it properly, many environmental aspects related to the functioning of cities need to be considered. In this context, an appropriately constructed synthetic measure that considers many assessment criteria can be a useful tool for analysis and evaluation. By determining the synthetic measure, it is possible to create a ranking of the cities included in the study. This then makes it possible to order cities from those which are characterised by a low level of environmental sustainability, to those which are in the best situation in that respect. The construction of such a measure first requires the selection of assessment criteria and sub-measures, and then bringing the sub-measures to comparable values.

The procedure of synthetic measure construction is determined by many factors. It depends on the nature of the diagnostic variables, the scale of their measurement, the standardisation procedure, the weighting system, and the aggregation formula of these variables. If the diagnostic variables are expressed in different measurement units and/or correspond to different numerical ranges, then they should be transformed to obtain variables without denominators and unified as to the range of values they can take. The literature [89,90] points to numerous methods of normalising diagnostic variables. One such method is the method of zeroed unitarisation, which makes it possible to normalise diagnostic variables based on examining the trait bifurcation. This method meets all the postulates posed for the procedures of normalising diagnostic variables. The resulting values of variables are contained in the range [0;1].

According to the method of zero unitarisation there is a constant reference point, which is the range of the normalized variable:

(1) $$R\left(X_{j}\right)=\underset{}{\underset{i}{\max}x_{ij}\;}-\underset{i}{\min}x_{ij}$$

Depending on the nature of the diagnostic variable, normalisation is carried out using the following formulas [91]:

(a) for stimulants, i.e., diagnostic variables, whose increase in value causes an increase in the assessment of a complex phenomenon:

(2) $$z_{ij}=\frac{x_{ij}-\underset{i}{\min}x_{ij}}{\underset{i}{\max}x_{ij}-\underset{i}{\min}x_{ij}},$$

(b) for destimulants, i.e., diagnostic variables, whose increase in value causes a decrease in the evaluation of a complex phenomenon:

(3) $$z_{ij}=\frac{\underset{i}{\max}x_{ij}-x_{ij}}{\underset{i}{\max}x_{ij}-\underset{i}{\min}x_{ij}},$$

(c) for nominants, i.e., diagnostic variables, which have a specific value, most favourable from the point of view of the evaluation of a complex phenomenon, called the nominal value:

(4) $$z_{ij}=\{\begin{array}{c} \frac{x_{ij}-\underset{i}{\min}x_{ij}}{c_{oj}-\underset{i}{\min}x_{ij}}\;for\;x_{ij}<c_{0j}\\ \;1\;for\;x_{ij}=c_{0j}\\ \frac{\underset{i}{\max}x_{ij}-x_{ij}}{\underset{i}{\max}x_{ij}-c_{0j}}\;for\;x_{ij}>c_{0j} \end{array}\;$$

For characteristics that are nominants, the following relationships occur:

(5) $$z_{ij}=1\underset{}{\Leftrightarrow }x_{ij}=c_{0j}$$

and

(6) $$z_{ij}=0\underset{}{\Leftrightarrow }x_{ij}=\underset{i}{\min}x_{ij}\;\mathrm{or}\;\;x_{ij}=\underset{i}{\max}x_{ij}$$

where:

*z_ij_*—a normalised diagnostic variable *i* for object *j* taking a value between [0;1];
*x_ij_*—the value of characteristic *i* in *j-*th object;
min*_i_ x_ij_*—the lowest value of characteristic *i* among the objects in the set [1, 2,…, *j*];
max*_i_ x_ij_*—the highest value of characteristic *i* among the objects in the set [1, 2,…, *j*];
*c_0j_—*nominal value of the characteristic at *j*-th object.

The next step is the construction of a synthetic measure based on the following formula:

(7) $$Q_{i}=\underset{j=1}{\sum }z_{ij}\omega_{j}$$

where weights *w_j_* satisfy the condition of summability to one:

(8) $$\underset{j=1}{\sum }\omega_{j}=1$$

The procedure presented was used to construct a synthetic measure relating to the assessment of the degree of environmental sustainability of European Union capital cities.

The set of diagnostic variables was proposed based on the literature research. An important premise for the selection of individual variables for the study was the availability of relevant statistical data. 

The selection of indicators for the study also results from the analysis of existing rankings of sustainable cities carried out in the section ‘Rankings and indicators of environmental sustainability’. It describes the most frequently used environmental indicators concerning air pollution, noise, green spaces, sustainable buildings, and electric car charging stations. Their relevance was justified. On this basis, they were included among the diagnostic variables in this study. Moreover, the cited section points out the need to combine quantitative and qualitative data in this type of research for greater comprehensiveness of results. This postulate was also reflected in our study. As a consequence, variables based on surveys of residents were used that concerned the degree of satisfaction with selected environmental aspects related to the functioning of the cities in which they live.

The set of potential diagnostic variables was subjected to selection due to substantive criteria, i.e., mainly their informative potential. Due to the purpose of the study and the nature of the diagnostic variables (variables relating to the scope of environmental sustainability may be naturally coupled), a restrictive approach to the selection of diagnostic variables based on formal criteria was abandoned. The set of diagnostic variables included the most recent available data on selected environmental aspects of city performance (Table 1).

The air quality diagnostic variables included pollutants regulated under the EU’s Air Quality Directives [92,93]:

- benzo(a)pyrene (BaP);
- nitrogen dioxide (NO_2_);
- ozone (O_3_);
- particulate matter (both PM2.5 and PM10).

The emission of pollutants within variables X_1_–X_5_ was calculated as an average result of all available measuring stations located within the cities. The principle of ‘the less the better’ was adopted, although the acceptable level is determined by global guidelines [94,95] in addition to European Union Directives.

A certain limitation in the research process was the availability of complete statistical data for all EU capital cities. The lack of complete data for three European capitals, such as Bucharest, Nicosia, Valletta, meant that these cities were eliminated from further analysis.

## 4. Results

Based on the procedure proposed above, the synthetic measure was constructed. The values of the synthetic indicator determined for all cities included in the analysis are presented in Table 2.

The synthetic measure also made it possible to create a typology of European capitals according to their level of environmental sustainability. This typology is based on a grouping method using the arithmetic mean and the standard deviation. Thus, the cities studied were divided into the following four groups: 

I—a group of European capitals with a very high level of environmental sustainability, for which the following condition is satisfied:

(9) $$Q_{i}\geq {\bar{Q}}_{i}+S(Q_{i})$$

II—a group of European capitals with a high level of environmental sustainability, for which the following condition is satisfied:

(10) $${\bar{Q}}_{i}\leq Q_{i}<{\bar{Q}}_{i}+S(Q_{i})$$

III—a group of European capitals with an average level of environmental sustainability, for which the following condition is satisfied:

(11) $${\bar{Q}}_{i}-S\left(Q_{i}\right)\leq Q_{i}<{\bar{Q}}_{i}$$

IV—a group of European capitals with a low level of environmental sustainability, for which the following condition is satisfied:

(12) $$Q_{i}<{\bar{Q}}_{i}-S(Q_{i})$$

where:

(13) $${\bar{Q}}_{i}=\frac{1}{n}\overset{n}{\underset{i=1}{\sum }}Q_{i}$$

(14) $$S\left(Q_{i}\right)=\sqrt{\frac{1}{n}\overset{n}{\underset{i=1}{\sum }}{\left(Q_{i}-{\bar{Q}}_{i}\right)}^{2}};\;\left(i=1,\;2\ldots n\right).$$

Based on a set of standardised diagnostic variables, the synthetic indicator *Q_i_* quantitatively measures the environmental sustainability of EU capital cities. The comparison made it possible to identify the leaders. These are mainly Nordic capitals, as well as capitals of northern European countries. The *Q_i_* index was particularly high for the following five capitals: Helsinki (0.713), Stockholm (0.699), Dublin (0.678), Amsterdam (0.667), and Tallinn (0.664). The analysis of the detailed data used as diagnostic variables in the study shows that these cities are mainly characterised by a good air quality situation, which is the result of continuously reduced emissions of pollutants into the atmosphere (values of standardised diagnostic variables are presented in Table A1 in the Appendix A). It is certainly influenced by many factors, such as use of clean technologies in industry, transport or municipal services, care of municipal authorities for green areas in the city, or generally any support for pro-environmental directions of city development. The study and the analysis of individual diagnostic variables show that the inhabitants of the capital cities ranked highest (group I) assess very well not only the quality of air but are also satisfied with the fact that the city is relatively clean, quiet, and does not lack green areas. Public transport is at a satisfactory level. There is also an alternative to public transport, e.g., cycling, which is supported by numerous cycle lanes.

Group II (see Table 2) includes the capital cities with a fairly high level of environmental sustainability. Copenhagen, with a *Q_i_* value of 0.611, was ranked highest in this group. This city, like other northern European capitals, is characterised by good air quality and environmentally friendly urban infrastructure. In group II, Paris was rated lowest (*Q_i_* = 0.486). This was a result of low scores for green space in the city and for environmentally friendly modes of transport, such as cycling.

The group of European capitals with an average level of environmental sustainability (Group III) includes mainly CEE capitals, but also Lisbon, Brussels, and Madrid. These cities have certain problems and limitations that affect the final rating and ranking. This includes not only poor air quality in the city but other elements such as noise or limited possibilities to use environmentally friendly means of transport.

Group IV, the lowest ranked capital cities, includes four southern European cities: Zagreb (*Q_i_* = 0.329), Athens (*Q_i_* = 0.252), Rome (*Q_i_* = 0.233), and Sofia (*Q_i_* = 0.222). These are cities with many environmental problems—noise, poor air quality, urban cleanliness problems, and relatively small green spaces. As these are capital cities of not very wealthy countries, underinvestment in environmental infrastructure is also visible. There are relatively few green buildings or public charging stations in these cities. The environmental problems of these cities are perceived not only by their inhabitants but also by tourists.

## 5. Discussion

The paper attempts to assess the level of environmental sustainability of EU capital cities. A ranking of cities was created based on diagnostic variables using quantitative and qualitative indicators relating to the quality of the environment in the studied cities. The literature indicates that urban sustainability indicators can be an effective instrument not only for assessing the performance of cities, but above all for improving it [78]. The analysis presented here, therefore, has some application potential and can be used by policy makers at different levels of urban management.

The results of our study show that, in addition to the group of Nordic cities, Dublin, Amsterdam, and Tallinn also recorded the best results in terms of environmental sustainability levels. Northern European cities clearly dominate southern European cities (Zagreb, Athens, Rome, and Sofia) in this respect. In order to identify the reasons for this state of affairs, it would be advisable to deepen the research and take into account economic and social factors. They are relevant since analyses by Brilhante and Klaas have shown that cities with high GDP have good performance in sanitation, air quality, waste, drinking water, and transport [13]. Also, historical considerations that have shaped the development capacity of individual countries influence the degree of environmental pollution [96]. Thus, it cannot be excluded that, in addition to the income aspect or historical considerations, the mentality of the population or the urban management strategy, for examples, have a key influence on the environmental situation.

The results obtained show a high degree of convergence with the results of other rankings that include environmental sub-indexes within the estimation of the performance of sustainable cities [54,55,61]. An example is the IESE Cities in Motion Index 2020, in which Copenhagen, Stockholm, and Helsinki are ranked highest among EU capitals, while Bucharest and Rome score the worst [97]. In more recent rankings, behavioural and travel restrictions related to the COVID-19 pandemic have had an impact on the results, as they reduced the amount of harmful gases in the air. This situation was considered in the environmental sub-index that is part of the Global Power City Index. Despite these changes, the best results were achieved by Stockholm, Copenhagen, Vienna, and Helsinki [98]. Very similar results were obtained in the environmental category of the Safe City Index 2021, in which the top three, apart from Copenhagen, Stockholm, were joined by Amsterdam [99]. This category is based on quantitative indicators, but also on the assessment of the efficiency of city management, including efficiency of environmental planning, taking green initiatives, and management of water resources. This is a certain novelty that distinguishes this index from others. When comparing the results of the above rankings with the rankings from previous years, some differences can be seen. For example, Tallinn, which is currently highly ranked, performed very poorly in the 2009 European Green City Index [51]. In turn, a significant regression in environmental performance concerned Madrid, which as recently as 2015 was at the top of the environmental subcategory in the Sustainable Cities Index 2015; meanwhile, today it performs much more poorly [100]. In such cases, it is difficult to identify what is causing the changes. The rankings produced by public and private institutions differ in every respect. Different sets of indicators, methods of standardisation, weighting factors, and methods of creating synthetic indices cause great complications in comparing results. Despite so many differences in measurement methods, the northern European capitals are always at the forefront of environmental performance.

The Nordic countries have been considered leaders in planning and implementing long-term, low carbon energy transitions strategies for several decades [101]. These are based on the use of hydropower, geothermal, wind, biofuels energy, and others. However, what clearly distinguishes northern countries from other European Union capitals is the higher willingness of residents to sacrifice economic well-being for the environment, manifested also in their pro-environmental preferences and behaviours [102]. This is evident in the greater popularity of bicycle transport, the use of public transport, or in the choice of ecological solutions in the construction of houses, etc. A noteworthy case among the leaders in our ranking is Tallinn. It is the city with the lowest concentration of particulate matter (for both PM2.5 and PM10) among all European capitals. This results from, for example, special programs of the city promoting public transport. The city authorities introduced a ‘green card’ for residents at a symbolic price, which allowed free access to the city’s bus, tram, trolleybus, and train network. This increased the use of urban public transport by 14% [103]. It is also worth mentioning that Estonians work closely with Finns to develop environmental innovations, which undoubtedly results in better environmental care.

Considering the spatial distribution of our results, it can be seen that the clean north of Europe clearly dominates the polluted South (Zagreb, Athens, Rome, and Sofia). These results are largely consistent with the SDG Index for European Cities, where a north-south divide also emerged [55]. Slightly different were the results of the Lisbon ranking for smart sustainable cities, where western Europe scored better than eastern Europe [33]. Such a division does not occur in our study. Indeed, the side of cities with the best results included Tallinn and also Riga, Vilnius, and Ljubljana. However, western cities such as Brussels, Madrid, and Rome were assigned to the two groups with the lowest level of environmental sustainability. Thus, this is another contribution to expanding research into the reasons for this division.

## 6. Conclusions

Cities all over the world must cope with the consequences of a growing population. Choosing the right strategy for their development is therefore crucial. The vast majority of contemporary development models, such as smart city, green city, sustainable city, and smart sustainable city, take environmental aspects into account. Sustainable urban systems ‘enable human settlements to survive and thrive with the least possible impact on natural systems’. The environment creates certain constraints for economic and social development; therefore, its protection and the reduction in negative human impact on it have become one of the most important objectives of the functioning of the European Union. Monitoring the state of the environment is therefore part of this strategy.

In this article, the research aim was to evaluate the level of environmental sustainability in capital cities of the European Union member states. In order to achieve the aim, it was necessary to answer the research questions posed. Based on the literature and environmental rankings of cities and countries, a set of indicators was selected that enabled a comparative assessment of European cities in terms of their level of environmental sustainability. Multidimensional comparative analysis was used in order to develop a ranking of cities, and to group them according to their level of environmental sustainability. The results of the study showed that the top-ranked cities were those in northern Europe, where environmental quality was rated the highest, and also according to their residents. The greatest challenges to improving environmental performance are faced by capital cities in the southern part of the European Union.

The contribution of the article can be perceived through the creation of a synthetic index reflecting the level of environmental sustainability of European capital cities and the construction of a ranking of the examined cities. The index is based on an indicator-based tool and can also be used to analyse other issues. The method proposed by us can be used in practice—by policymakers responsible for the implementation of environmental policy in metropolitan areas. Moreover, the analysis of the values of particular diagnostic variables for each city may provide interesting information on areas to which particular attention should be paid when undertaking investments related to urban development.

However, it is worth noting that the presented study has some limitations. It does not consider other aspects of environmental assessment, such as water quality, solid waste treatment, energy consumption, renewable energy production, etc. This is related to the difficulty of accessing up-to-date public, reliable, and comparable data concerning the examined cities. Therefore, there is a need to deepen this type of research by considering additional diagnostic variables. It would also be worth making cyclic measurements to identify the dynamics of changes in the level of environmental sustainability of EU cities. Interesting results could also be obtained from studies verifying relations between the level of environmental sustainability of cities and the quality of life of their inhabitants, or their general level of life satisfaction. Indeed, it appears that cities that improve their sustainability performance may have a greater impact on the happiness and well-being of their inhabitants. This is undoubtedly an interesting direction for further research, the results of which may generate applied lessons for urban development policy.

It should be noted that the results of analyses conducted using the methods of multivariate comparative analysis may differ in the case of adopting a different set of diagnostic variables and a different system of weights. Objectivisation of this type of research would certainly be facilitated by relying on a fixed, universally accepted set of diagnostic variables. However, such a set has not been developed so far. Our research was also influenced by the limited access to reliable and comparable data at the local level. A certain limitation in the process of assessing the sustainability of cities and other spatial units is also the fact that there is no so-called model city which could be used as a reference point for the assessment of the level of environmental sustainability. It is worth noting that there will always be room for improvement in the environmental situation of each city.

## Figures and Tables

**Table 1:** Set of diagnostic variables.

Diagnostic Variable	Additional Explanations	Source
X_1_—Annual average BaP emissions ng/m^3^	-	European Environment Agency, 2019
X_2_—Annual average NO_2_ emissions ug/m^3^	-	
X_3_—O_3_ emissions ug/m^3^	The 93.15th percentile of daily 8 h maximum in a given year	
X_4_—Annual average PM2.5 emissions ug/m^3^	-	
X_5_—PM10 emissions ug/m^3^	The 90.41th percentile of daily averages in a given year	
X_6_—Green buildings *per square kilometre*	LEED-certified buildings	U.S. Green Building Council (USGBC), 2021
X_7_—Public charging stations for electric cars *per square kilometre*	-	Chargemap, 2021
X_8_—Tree cover, percent of total functional urban area	-	OECD.STAT, 2019
X_9_—CO_2_ emission index	An estimation of CO_2_ consumption due to traffic time	Numbeo, 2021
X_10_—Green spaces such as public parks or gardens: very satisfied and rather satisfied, percentage	-	Eurostat, Perception Survey Results, 2019
X_11_—Sports facilities such as sport fields and indoor sport halls in the city: very satisfied and rather satisfied, percentage	-	
X_12_—Means of transport most often used: bicycle and/or foot, percentage	-	
X_13_—The quality of the air in the city: very satisfied and rather satisfied, percentage	-	
X_14_—The noise level in the city: very satisfied and rather satisfied, percentage	-	
X_15_—The cleanliness in the city: very satisfied and rather satisfied, percentage	-	

**Table 2:** Ranking of European Union capitals according to their level of environmental sustainability.

Ranking	Group	City	*Q_i_*
1	I	Helsinki	0.713
2		Stockholm	0.699
3		Dublin	0.678
4		Amsterdam	0.667
5		Tallinn	0.664
6	II	Copenhagen	0.611
7		Luxembourg	0.602
8		Vienna	0.578
9		Riga	0.546
10		Vilnius	0.533
11		Ljubljana	0.500
12		Berlin	0.498
13		Paris	0.486
14	III	Lisbon	0.463
15		Brussels	0.439
16		Prague	0.438
17		Madrid	0.382
18		Bratislava	0.372
19		Budapest	0.349
20		Warsaw	0.343
21	IV	Zagreb	0.329
22		Athens	0.252
23		Rome	0.233
24		Sofia	0.222

## Data Availability

Data supporting reported results can be obtained by contacting the authors. Requests for access to these datasets should be directed to Justyna Łapińska: justlap@umk.pl.

## Appendix A

**Table A1:** Values of standardised diagnostic variables.

Specification	X_1_	X_2_	X_3_	X_4_	X_5_	X_6_	X_7_	X_8_	X_9_	X_10_	X_11_	X_12_	X_13_	X_14_	X_15_
Amsterdam	0.791	0.435	0.743	0.508	0.697	0.300	1.000	0.000	0.796	0.935	0.864	1.000	0.656	0.639	0.641
Athens	0.985	0.174	0.297	0.144	0.201	0.998	0.068	0.124	0.056	0.000	0.000	0.441	0.037	0.000	0.256
Berlin	0.816	0.180	0.119	0.368	0.677	0.097	0.122	0.616	1.000	0.920	0.488	0.379	0.717	0.511	0.465
Bratislava	0.787	0.613	0.000	0.346	0.556	0.050	0.024	0.351	0.772	0.406	0.217	0.250	0.434	0.509	0.267
Brussels	0.928	0.324	0.564	0.422	0.675	0.411	0.185	0.010	0.089	0.876	0.662	0.254	0.399	0.404	0.382
Budapest	0.483	0.338	0.531	0.284	0.225	0.124	0.055	0.215	0.586	0.640	0.405	0.254	0.371	0.315	0.407
Copenhagen	0.885	0.710	0.677	0.535	0.611	0.250	0.004	0.176	0.924	0.970	0.490	0.873	0.651	0.682	0.732
Dublin	0.955	0.550	0.997	0.672	0.927	1.000	0.097	0.030	0.320	0.914	0.841	0.305	0.931	1.000	0.634
Helsinki	0.783	0.719	0.703	0.897	0.698	0.000	0.061	0.834	0.908	1.000	1.000	0.342	1.000	0.906	0.835
Lisbon	0.960	0.503	0.648	0.562	0.723	0.328	0.205	0.318	0.296	0.605	0.438	0.186	0.424	0.356	0.392
Ljubljana	0.212	0.537	0.223	0.149	0.476	0.001	0.055	0.702	0.466	0.920	0.848	0.529	0.783	0.697	0.904
Luxembourg	0.890	0.393	0.488	0.608	0.759	0.026	0.170	0.489	0.341	0.946	0.942	0.276	0.876	0.830	1.000
Madrid	0.937	0.153	0.207	0.599	0.737	0.612	0.027	0.283	0.218	0.610	0.353	0.279	0.101	0.264	0.352
Paris	0.919	0.083	0.549	0.390	0.491	0.745	0.760	0.251	0.648	0.868	0.517	0.270	0.059	0.335	0.404
Prague	0.439	0.443	0.154	0.254	0.506	0.141	0.015	0.328	0.842	0.790	0.924	0.162	0.550	0.446	0.570
Riga	0.738	0.577	1.000	0.450	0.351	0.023	0.015	1.000	0.422	0.889	0.360	0.171	0.685	0.743	0.767
Rome	0.663	0.000	0.409	0.371	0.406	0.036	0.011	0.288	0.000	0.475	0.423	0.057	0.096	0.262	0.000
Sofia	0.389	0.444	0.795	0.000	0.089	0.010	0.000	0.140	0.499	0.553	0.018	0.000	0.000	0.096	0.298
Stockholm	1.000	0.664	0.484	0.917	0.540	0.754	0.071	0.953	0.785	0.979	0.465	0.478	0.836	0.831	0.732
Tallin	0.903	1.000	0.714	1.000	1.000	0.157	0.044	0.438	0.692	0.892	0.626	0.147	0.823	0.695	0.826
Vienna	0.777	0.650	0.069	0.416	0.706	0.083	0.194	0.271	0.954	0.963	0.613	0.320	0.944	0.790	0.918
Vilnius	0.679	0.738	0.487	0.348	0.547	0.029	0.002	0.644	0.633	0.890	0.358	0.311	0.733	0.775	0.816
Warsaw	0.066	0.379	0.402	0.005	0.227	0.146	0.007	0.365	0.588	0.865	0.649	0.156	0.350	0.268	0.677
Zagreb	0.000	0.215	0.178	0.176	0.000	0.002	0.006	0.455	0.468	0.833	0.450	0.138	0.682	0.631	0.709
